# Multi-omic detection of *Mycobacterium leprae* in archaeological human dental calculus

**DOI:** 10.1098/rstb.2019.0584

**Published:** 2020-10-05

**Authors:** Anna K. Fotakis, Sean D. Denham, Meaghan Mackie, Miren Iraeta Orbegozo, Dorothea Mylopotamitaki, Shyam Gopalakrishnan, Thomas Sicheritz-Pontén, Jesper V. Olsen, Enrico Cappellini, Guojie Zhang, Axel Christophersen, M. Thomas P. Gilbert, Åshild J. Vågene

**Affiliations:** 1 Section for Evolutionary Genomics, GLOBE Institute, Faculty of Health and Medical Sciences, University of Stavanger, Stavanger, Norway; 2 Museum of Archaeology, University of Stavanger, Stavanger, Norway; 3 Novo Nordisk Foundation Centre for Protein Research, University of Copenhagen, Copenhagen, Denmark; 4 Section for Ecology and Evolution, Department of Biology, University of Copenhagen, 2100 Copenhagen, Denmark; 5 BGI-Shenzhen, 518083 Shenzhen, People's Republic of China; 6 State Key Laboratory of Genetic Resources and Evolution, Kunming Institute of Zoology, Chinese Academy of Sciences, 650223 Kunming, People's Republic of China; 7 Centre for Excellence in Animal Evolution and Genetics, Chinese Academy of Sciences, 650223 Kunming, People's Republic of China; 8 NTNU University Museum, Trondheim, Norway

**Keywords:** ancient DNA, palaeoproteomics, leprosy, *Mycobacterium leprae*, dental calculus

## Abstract

Mineralized dental plaque (calculus) has proven to be an excellent source of ancient biomolecules. Here we present a *Mycobacterium leprae* genome (6.6-fold), the causative agent of leprosy, recovered via shotgun sequencing of sixteenth-century human dental calculus from an individual from Trondheim, Norway. When phylogenetically placed, this genome falls in branch 3I among the diversity of other contemporary ancient strains from Northern Europe. Moreover, ancient mycobacterial peptides were retrieved via mass spectrometry-based proteomics, further validating the presence of the pathogen. *Mycobacterium leprae* can readily be detected in the oral cavity and associated mucosal membranes, which likely contributed to it being incorporated into this individual's dental calculus. This individual showed some possible, but not definitive, evidence of skeletal lesions associated with early-stage leprosy. This study is the first known example of successful multi-omics retrieval of *M. leprae* from archaeological dental calculus. Furthermore, we offer new insights into dental calculus as an alternative sample source to bones or teeth for detecting and molecularly characterizing *M. leprae* in individuals from the archaeological record.

This article is part of the theme issue ‘Insights into health and disease from ancient biomolecules’.

## 1. Introduction

**Figure 1.**
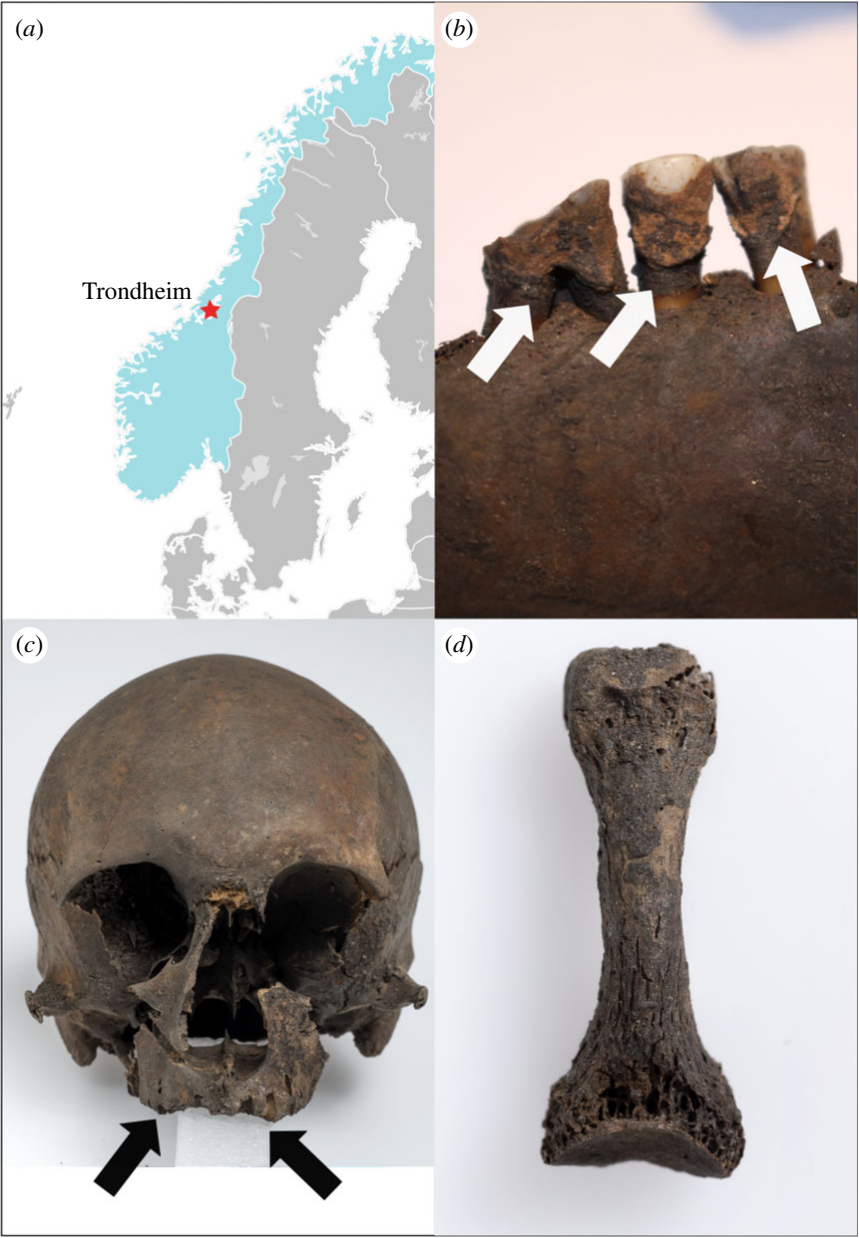
(*a*) Location of Trondheim, Norway; (*b*) dental calculus found from SK92 (white arrows indicate location) (photo: Anna Fotakis); (*c*) evidence of rhinomaxillary syndrome, with possible loss of upper central incisors and resorption of the bone of the associated alveoli (indicated by black arrows); (*d*) concentric loss of bone from pedal phalanx. Photos (*c*,*d*): Åge Hojem, NTNU University Museum; arrows in (*c*) were added by the authors. (Online version in colour.)

Mineralized dental plaque (calculus) from the archaeological record is a rich reservoir of ancient biomolecules [1], containing endogenous oral microbiota, opportunistic pathogens and food particles [2,3]. Since the original application of next-generation sequencing to archaeological calculus [4], a number of papers have documented its use in recovering host genomic and metagenomic sequences [5,6], as well as proteomic [3,7] and metabolomic [8] information, emphasizing the unique preservation potential of this substrate. These studies have predominantly focused on oral microbiota and oral pathogens, and only a few have focused on evidence for calculus containing pathogens that are acquired, rather than commensal in origin, and are, therefore, not a standard component of the human oral microbiome [9]. Here, we demonstrate that it is possible to retrieve genomic DNA and peptides belonging to *Mycobacterium leprae* (*M. leprae*), an acquired pathogen, from the calculus of a sixteenth-century individual from Trondheim, Norway (figure 1*a*).

Leprosy, a chronic granulomatous infection caused by *M. leprae*, was prevalent across Europe during the medieval period until its perceived decline on the mainland in the sixteenth century [10,11]. However, this was not the case in Norway where leprosy increased in prevalence well into the nineteenth century [12,13]. The reason for leprosy's persistence in Norway is unclear, but hypotheses posit that it may have been strengthened by poor housing conditions and peaty soils [12,13].

*Mycobacterium leprae* is one of few pathogens where prolonged infection can cause distinctive bone lesions to form during the advanced stages of disease progression. Therefore, skeletal remains displaying such lesions have been targeted by previous studies seeking to retrieve ancient *M. leprae* DNA [14–17]. However, the number of visually identifiable individuals infected with *M. leprae* at the time of death is limited, because it is not possible to account for individuals that died before bone lesions could form, or where lesions cannot definitively be attributed to leprosy, or where remains are poorly preserved [18].

In this study, we recovered a complete *M. leprae* genome (6.6-fold average coverage), as well as a number of peptides assigned to the *Mycobacterium* genus, from dental calculus of an individual radiocarbon dated to the sixteenth century from Trondheim, Norway. The individual displayed lesions affecting the rhinomaxillary cavity and foot bones indicative of the early stages of leprosy. However, these lesions are not pathognomonic and, therefore, differential diagnoses could not be ruled out.

This is the first study, to our knowledge, where both proteomic and DNA sequence data have been used to detect and validate the presence of *M. leprae* from dental calculus. Using shotgun metagenomic data, we also provide a comparison of *M. leprae* DNA preservation in both dental calculus and tooth-root dentine.

## 2. Material and methods

### (a) Sampling strategy and osteological analyses

Dental calculus (figure 1*b*) and a premolar tooth belonging to a female individual (SK92/N86160) excavated from the ‘Library site’ in Trondheim were sampled at NTNU Trondheim for metagenomic and proteomic analysis. Calculus was sampled on clean small measuring boats placed on foil strips using sterilized dental equipment. Additionally, a 1.2 g sample of bone from the posterior surface of the left femur was taken for radiocarbon dating and stable isotopic analysis. Both tests were performed at the National Laboratory for Age Determination, NTNU. Sex was estimated according to the standards in Buikstra & Ubelaker [19], age-at-death using transition analysis [20] in the ADBOU age estimation software package and skeletal pathology recorded following Waldron [21].

### (b) Ancient DNA extraction and library preparation

All pre-amplification laboratory preparations were carried out in dedicated ancient biomolecules clean laboratory facilities at the GLOBE Institute, University of Copenhagen. For every step described below, negative controls (tubes with no calculus) underwent the same procedure.

#### (i) Calculus

DNA extractions for both dentine and calculus followed a modified silica-in-solution protocol as described in Allentoft *et al.* [22]. Thirty-five milligrams of calculus were used for DNA extraction. Approximately 60 ng of DNA extract was used to generate a double-stranded library according to the BEST protocol [23] using a modified adapter sequence suitable for BGISeq500 [24]. Quantitative real-time PCR (qPCR) with 2X Roche LC480 MasterMix (KAPA biosystems) was used to estimate the required number of cycles for library index PCR amplification (17 cycles for both dentine and calculus). Post-qPCR, library index amplifications were performed in a 50 µl reaction using BGI indexes and the KAPA HiFi HotStart Uracil+ ReadyMix PCR protocol (KAPA Biosystems). After this, libraries were purified with QiaQuick columns (Qiagen) and eluted with 30 µl buffer EB (Qiagen). Quantification and fragment size estimation was done using the High-Sensitivity DNA Assay for the Bioanalyzer2100 (Agilent). The sample library and its associated negative controls were paired-end sequenced at BGI Shenzhen (2 × 50 bp) using the BGISeq500 platform. Two hundred and eighty-one million reads were generated for the calculus library and 1–7 million for the negative controls.

#### (ii) Dentine

Two hundred and fifty milligrams of dentine drilled from the tooth root were extracted as described above for the calculus. A double-stranded library was generated using 32 µl of extract according to the BEST protocol, using adapters compatible with Illumina sequencing according to Carøe *et al*. [23]. qPCR and PCR reactions were prepared using Amplitaq Gold (ThermoFisher) and purified in 30 µl using SPRI beads as in Rohland & Reich [25]. The amplified library was single-end sequenced with Illumina 2500 (1 x 80 bp) at the Danish National High-throughput DNA Sequencing Centre. A total of 3.6 million single-end reads were generated.

### (c) Initial read processing for SK92 and negative controls

The paired-end BGI reads were de-multiplexed as described previously by Mak *et al.* [24]. Adapters were removed from all samples and negative control libraries using Adapter Removal v. 2.2.4 [26], paired-end reads were collapsed. Only collapsed reads were used in downstream analysis for all paired-end data.

### (d) Taxonomic screening

To confirm that the taxonomic profile is consistent with oral samples, Metaphlan2 was used to create a metagenomic profile of the non-human dental calculus reads. The reads were aligned to the MetaPhlan2 database [27] using Bowtie2 v. 2.2.9 [28] and PCR duplicates were excised using the filteruniquebam tool in PALEOMIX [29]. We compared the obtained taxonomic profile of abundances with 689 human microbiome profiles published from the Human Microbiome Project (HMP) Consortium [30], comprising samples from the mouth (*N* = 382), skin (*N* = 26), gastrointestinal tract (*N* = 138), urogenital tract (*N* = 56), airways and nose (*N* = 87). The oral HMP samples consist of attached/keratinized gingiva (*N* = 6), buccal mucosa (*N* = 107), palatine tonsils (*N* = 6), tongue dorsum (*N* = 128), throat (*N* = 7), supragingival plaque (*N* = 118) and subgingival plaque (*N* = 7). We compared pairwise ecological distances, the difference in species composition, among all profiles at genus and species level using taxon relative abundances and the vegdist function from the vegan package in R [31]. These were used for principal coordinate analysis (PCoA) of the Bray–Curtis distances in R using the pcoa function included in the APE package [32] (electronic supplementary material, figure S1). We calculated the average relative abundance for each genus and for each body site present in the HMP and visualized the abundance of the top 50 genera present using hclust2 (https://bioconda.github.io/recipes/hclust2/README.html) by hierarchically clustering (average linkage) with the Bray–Curtis similarity (electronic supplementary material, figure S2).

### (e) Pathogen screening

Reads from the SK92 sample libraries and negative controls were screened for the presence of pathogen DNA with the metagenomic analysis tool MALT v. 0.4.1 [33], using a custom taxonomic database. The database comprised all complete bacterial (*n* = 12 427) and viral (*n* = 8096) genomes downloaded from NCBI RefSeq on 13 February 2019, and all complete archaeal (*n* = 280) genomes downloaded from NCBI RefSeq on 17 April 2019. The following two entries were excluded: ‘GCF_000954235.1 uncultured phage WW-nAnB’ and ‘GCF_000146025.2 uncultured Termite group 1 bacterium phylotype Rs-D17’, because they are uncultured and derive from complex metagenomic datasets, and may, therefore, consist of composite sequences from multiple organisms.

The final database contained 20 803 genomes; default parameters were used when creating it with malt-build (v. 0.4.1). The output ‘rma6’ files were manually inspected in MEGAN6 [34]. MALT results are shown in electronic supplementary material, table S1.

### (f) Mapping approach and dataset preparation

#### (i) SK92 and negative controls

The EAGER pipeline v. 1.92.55 [35] was used to map reads to the *M. leprae* TN reference genome (NC_002677.1) using Burrows–Wheeler Aligner v0.7.17 (bwa-aln) [36], remove duplicates using Picard tools v. 2.18.26 MarkDuplicates (http://broadinstitute.github.io/picard/), execute mapDamage v. 2.2.4 [37] and evaluate the data with Qualimap v. 2.2.2 [38]. Sensitive mapping parameters were used with bwa-aln (−*n* 0.01, −*l* 16) to accommodate deaminated bases. Mapquality (−*q*) 37 was used with samtools v. 1.3.1 [39]. The *M. leprae* reads from the SK92 calculus sample were mapped a second time as described above, although using more stringent mapping parameters with bwa-aln (−*n* 0.1 −*l* 32) to reconstruct the genome for downstream phylogenetic analyses. This was done due to the relatively low number of *M. leprae* reads with deaminated bases (electronic supplementary material, table S2), and the minimal loss of average coverage across the genome (see electronic supplementary material, tables S2 and S3). SNP calling was carried out with GATK v. 3.5.0 UnifiedGenotyper [40] for both the stringently and sensitively mapped reads for the SK92 calculus sample. The evaluation of the multiallelic variants present in the two genomes showed that the stringent mapping parameters also reduced the number of multiallelic SNPs infringing on the cut-off for homozygous calls. MultiVCFAnalyzer v. 0-85-1 [41] (https://github.com/alexherbig/MultiVCFAnalyzer) was used to compare the multiallelic variants called for the two mapping versions of the SK92 genome where a variant had to be covered by a minimum of five reads and have a minimum variant quality score of 30. Variants with allele frequency between 10 and 90% were classified as multiallelic (two or more conflicting base calls). Variants were determined to be homozygous if at least 90% of the reads agreed. The allele frequencies for two-state multiallelic variants were plotted in R [42] (electronic supplementary material, figure S3).

#### (ii) Processing of published modern and ancient *Mycobacterium leprae* genomes

The dataset for the phylogenetic analysis consisted of 164 previously published ancient and modern genomes [14–17,43–50]. This dataset was adapter clipped using AdapterRemoval v. 2.2.4 [26]. For paired-end data, reads were collapsed, and both collapsed and un-collapsed reads were used in downstream analyses. In cases where single-end and paired-end data existed for the same sample, the data were merged after separate adapter removal. Mapping and SNP calling were carried out using the EAGER pipeline as described above. In the dataset, all previously published ancient genomes were UDG-treated (deaminated bases removed), therefore, higher stringency parameters (−*n* 0.2, −*l* 32) were applied to the whole dataset (modern and ancient) when mapping with bwa-aln. This was done for all genomes with the exception of samples I30_W-09, NHDP-55, NHDP-63, NHDP-98, Thai-53 and 2936 that had an average read length below 50 bp (electronic supplementary material, table S3). Due to the shortness of these reads −*n* 0.1 was used instead, since −*n* 0.2 did not allow any mismatches to occur in many shorter reads and led to the loss of phylogenetic diversity.

Reference genomes Br4923 (NC_011896.1), Kyoto-2 (NZ_AP014567.1), MRHRU-235-G (NZ_CP029543.1) and TN (NC_002677.1) were included in the dataset by turning them into artificial sequence data (150 bp long reads with 2 bp tiling). They were fragmented using the Pyfasta 0.5.2 module in Python and subsequently converted into artificial fastq data. These reads were mapped using stringent parameters (−*n* 0.2 −*l* 32) with bwa-aln. The *Mycobacterium lepromatosis* (GenBank JRPY00000000.1) [51] genome was also processed in this way and used as the outgroup. It was mapped with sensitive bwa-aln parameters (−*n* 0.01 −*l* 16) to accommodate sequence divergence between *M. lepromatosis* and *M. leprae*.

#### (iii) Phylogenetic placement of SK92

**Figure 2.**
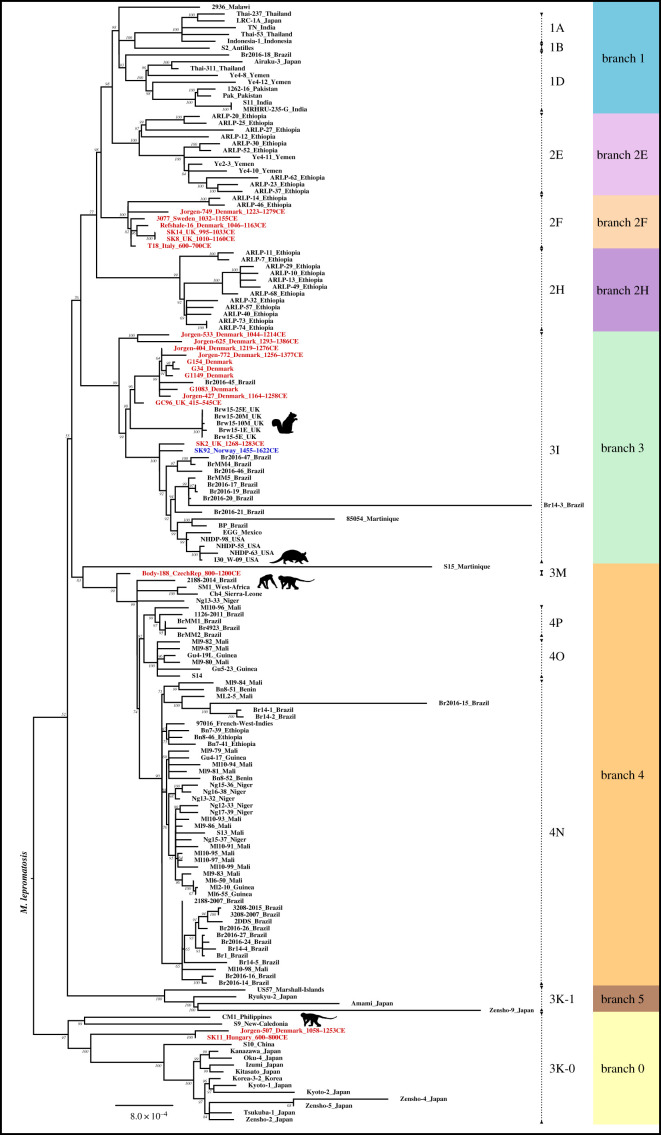
Maximum-likelihood tree of ancient and modern *M. leprae* strains. The tree is based on 3126 variant positions using 80% partial deletion and 1000 bootstrap replicates (bootstrap support for each node is shown). *Mycobacterium lepromatosis* was used as an outgroup. SK92 is coloured blue and previously published ancient genomes are coloured red. Radiocarbon dates and country of origin are provided for the ancient genomes where possible. The country of origin is provided for the modern genomes. Animal figures denote strains isolated from non-human primates, armadillos and squirrels. The primary branches are colour coded and the dotted lines indicate established SNP types (1–4) and subtypes (A–P). (Online version in colour.)

A multi-genome SNP alignment of homozygous positions for the dataset, including the stringently mapped SK92 calculus genome, was created using MultiVCFAnalyzer v. 0-85-1 [41]. Positions were classified as homozygous as described above, with the exception that five reads had to cover a position for it to be considered. Variant calls occurring in repetitive regions, rRNA and tRNA regions were excluded. The phylogenetically relevant position C251T in the *rrs* gene was not excluded [52]. A bone sample from skeleton SK12 from Winchester, UK—negative for *M. leprae*—harbouring contaminant DNA from soil-dwelling mycobacteria has been used in previous studies as a ‘negative control’ [14,15]. Thus, we also excluded SNPs called at 3X in SK12, when using sensitive bwa-aln mapping parameters (−*n* 0.01, −*l* 16). A list of excluded regions is provided in electronic supplementary material, table S4. The resulting SNP alignment contained 3139 variant positions in total. Eighty per cent partial deletion was applied, and the remaining 3126 variant positions were used to generate a maximum-likelihood phylogenetic tree using RAxML v. 8.2.11 [53] (figure 2). A model selection test was performed using MEGA X [54]; the GTR model performed best with the given dataset and was executed with RAxML using the ASC_GTRCAT model without rate heterogeneity (−V) using standard ascertainment bias correction and 1000 bootstrap replicates. Homozygous SNPs called for SK92 (SNPs in excluded regions not included) are listed in electronic supplementary material, table S5; SNP positions were annotated using snpEff v. 3.1 [55].

#### (iv) Human read analysis

The adapter-removed reads for the SK92 calculus and dentine (described above) were mapped to the human reference genome (hg19) using bwa-aln (v. 0.7.15) [36] (with −*n* 0.01, −*l* 1000). Reads with mapping quality lower than 30 were discarded and PCR duplicate reads were removed using Picard tools 2.13.2 (http://picard.sourceforge.net). Finally, reads were realigned around indels using Genome Analysis Toolkit3.3 [40] and the MD-tag was recalculated using samtools 1.6 [39]; mapDamage2.0 [37] was used to quantify post-mortem DNA damage. Summary statistics (depth of coverage, average read length and GC content) were estimated on the final dataset alignments using samtools 1.6 [39]. Mapping results were evaluated using Qualimap v. 2.2.2 [38].

#### (v) Normalization of damage pattern estimation

To accurately compare the deamination rates between the reads mapping to the *M. leprae* genome and the human genome in the SK92 calculus and dentine sequence data, the number of mapped reads was normalized. Picard tools was used to convert to fastq files and SeqKit [56] was used to extract 4000 mapping reads from the *M. leprae* alignment and 100 000 reads from the human genome alignments for SK92 calculus and dentine, respectively, with mapDamage2.0 [37] used to quantify post-mortem DNA damage for each subset. A summary of the deamination patterns can be found in electronic supplementary material, table S6.

### (g) Proteomics

#### (i) Ancient protein laboratory preparation

Protein extraction was undertaken in dedicated ancient biomolecules laboratories at the GLOBE Institute, University of Copenhagen. Dental calculus samples were placed in Protein LoBind tubes (Eppendorf) and weighed. Negative controls were tubes with no calculus sample. The protein extraction protocol used is described in depth in Jersie-Christensen *et al.* [7]. Prepared in-house C18 StageTips with immobilized samples were delivered to the Novo Nordisk Foundation Centre for Protein Research, University of Copenhagen, for further analysis by nanoflow liquid chromatography tandem mass spectrometry (LC-MS/MS) using a Q-Exactive HF mass spectrometer as described in Demarchi *et al*. [57], ‘Copenhagen setup’ and Kelstrup *et al*. [58].

#### (ii) Processing of metaproteomic data

Raw LC-MS/MS files of the sample and the extraction negative control were processed together in a single run by MaxQuant (v. 1.5.3.30) [59] using default Orbitrap settings. Search parameters included a fixed modification of carbamidomethylation for cysteine (C), and variable modifications for methionine oxidation (M) and deamidation (N,Q). Database searches were performed using the Andromeda search engine [60] against a concatenated FASTA file of the human reference proteome, entire SwissProt [61] and the Human Oral Microbiome Database (HOMD) [62], as described in Belstrøm *et al.* [63], retrieved August 2014 and applying a 1% FDR cut-off. The precursor mass tolerance was set to 5 ppm and the fragment mass tolerance was set to 20 ppm. Enzyme specificity was set to ‘Trypsin’ with one missed cleavage allowed and the minimum score for unmodified peptides was set to 40. Resulting protein and peptide identification were filtered for potential *Mycobacterium* identifications. All peptides of interest were then searched in BLASTp against the NCBI database (https://blast.ncbi.nlm.nih.gov/Blast.cgi) to validate species identifications and further validated in MQ viewer for spectrum inspection. The recovered peptides diagnostic for species identification are found in electronic supplementary material, table S7.

## 3. Results

### (a) Osteological analysis

Osteological evidence suggests that SK92 was a female adult aged 40–60 years. She had low quantities of dental calculus, evidence of small carious lesions and heavy tooth wear. Waldron's [21] operational definition for the identification of leprosy in skeletal remains requires the presence of either rhinomaxillary syndrome or concentric loss of bone on the manual or pedal phalanges. Rhinomaxillary syndrome involves several possible changes to the maxilla and the bones of the nose. These include the degeneration of both the anterior nasal spine and the maxilla (upper jaw) in the area of the incisors (leading to the loss of the upper incisors) as well as a rounding/widening of the nasal aperture [64]. In a living individual, this would appear as an inward collapse of the nose and upper jaw. SK92 does show several of these changes, although none to a strong degree. The alveolar process is resorbing, and the central incisors appear to have been lost pre-mortem with full alveolar resorption (figure 1*c*). There is some slight remodelling of the inferior margin of the nasal aperture. The anterior nasal spine and much of the nasal septum are missing, although it is unclear whether this is pathological or taphonomic in nature. One pedal phalanx shows some concentric loss of bone (figure 1*d*). Although all these changes seem to fulfil the operational definition, a conclusive diagnosis of leprosy requires more and stronger evidence, for instance, a much more clear-cut case of rhinomaxillary syndrome or several hand/foot elements with a more severe concentric loss of bone. From an osteological perspective, the conclusion must be probable early-stage leprosy.

### (b) Isotopic analysis and radiocarbon dating

The dating sample from SK92 (TRa-13412) returned a ^14^C age of 411 ± 14, rounded to 410 ± 15. However, differences between the terrestrial and marine carbon cycles can lead to the ^14^C ages of individuals with high marine dietary components appearing older than their actual age (i.e. the marine reservoir effect). The ^13^C ratio, *δ*13C (‰), in a sample allows for an estimation of the percentage marine dietary input, based on expected ^13^C ratios from 100% terrestrial and marine inputs, which can then be used to correct for this effect. Previous work on medieval Norwegian material has suggested terrestrial and marine dietary endpoints of −22‰ and −13‰, respectively [65]. In the present case, the stable isotopic results for ^13^C (*δ*13C = − 21.02‰) suggest a low-level marine protein contribution to the individual's diet, equating to a 10.9% marine dietary component (−22‰ and −13‰ were used as terrestrial and marine dietary end points, respectively, in the calculation). The ^14^C date was calibrated and corrected for the marine reservoir effect using the IntCal13 and Marine13 calibration curves [66] in Oxcal v. 4.3.2 [67]. The probability distribution of the calibrated, reservoir corrected date, reported at 2*σ*, has two age ranges: 1455–1524 CE (70.5%), 1590–1622 CE (24.9%). The median of the probability distribution is 1507 CE.

### (c) Pathogen screening

MALT [33] analysis of the metagenomic shotgun data generated from individual SK92's calculus (148 679 638 BGI input reads) and tooth-root dentine (35 980 654 Illumina input reads) revealed the presence of *M. leprae* in both sample libraries, where 1 484 427 and 4595 reads were assigned to *M. leprae* for the calculus and dentine, respectively (electronic supplementary material, table S2). Of the total reads assigned to the whole *Mycobacterium* genus, 99.0% and 96.8% of the calculus and dentine reads, respectively, correspond specifically to *M. leprae*. The high representation of *M. leprae* reads out of the total reads assigned to the genus *Mycobacterium* as a whole indicates that both the calculus and tooth-root-dentine samples suffered little contamination from soil-dwelling mycobacteria. The slightly higher percentage of non-leprae mycobacterial DNA in the tooth-root dentine may indicate that the calculus offered more protection against contaminating environmental sources. No reads from the negative controls were assigned to *M. leprae* in the MALT analysis (electronic supplementary material, table S1 and S2).

### (d) Genome reconstruction and phylogenetic placement

A 6.6-fold *M. leprae* genome was recovered from the dental calculus, where 76% of the genome was covered at least 5-fold (electronic supplementary material, table S2).

SK92 was phylogenetically placed within a dataset of previously published 20 ancient and 144 modern genomes. SK92 is positioned in branch 3I, forming a three-pronged multifurcation together with another ancient genome (SK2) and a branch consisting of modern genomes isolated from humans across Latin America, South America and southwestern USA [49,68], as well as an armadillo strain (I30_W-09) from the latter region [68]. The SK2 genome is from Winchester, UK, and is radiocarbon dated to the thirteenth century [14]. A further 10 medieval genomes, nine from Denmark and one from the UK, fall in the wider context of branch 3I (figure 2).

### (e) Metaproteomic signal of *Mycobacterium*

In the dental calculus sample, we identified four endogenous proteins, absent in the negative controls, that were assigned to the *Mycobacterium* genus (table 1). Most active genes are common among mycobacterial species, but a small subset of 136 genes are unique to *M. leprae* [69]. In our dataset, only three proteins were supported by peptides found in *M. leprae* exclusively, all of which were primarily related to virulence, detoxification and adaptation (electronic supplementary material, table S7). Many of the obtained peptides are shared among mycobacteria and we independently validated the results in order to avoid false positives [70] (table 1). However, taking into consideration from the MALT results that 99% of all *Mycobacterium* genus DNA reads found in the calculus were exclusively of *M. leprae* origin rather than a different mycobacterium, we conclude that the remaining protein is in fact from *M. leprae* as well.

**Table 1.** Mycobacterial proteins of the genus *Mycobacterium* detected within calculus sample SK92.

protein name	all matching peptides	unique peptides	total sequence coverage (%)	unique sequence coverage (%)	sequence length (aa)	function
60 kDa chaperonin 2	18	4	33.6	11.1	541	virulence detoxification and adaptation
18 kDa antigen	2	2	12.8	9	148	virulence detoxification and adaptation
bacterioferritin	3	3	24.5	24.5	159	intermediary metabolism and respiration
alkyl hydroperoxide reductase	2	1	7.2	3.6	195	virulence, detoxification and adaptation

### (f) *Mycobacterium leprae* and human endogenous DNA content comparison in dentine and calculus

Endogenous human DNA found within the dentine and the calculus followed a similar deamination pattern to that previously observed by Mann *et al.* [71]. In particular, the dentine sample had significantly higher deamination rates even when normalizing the number of reads. By contrast, *M. leprae* DNA deamination rates were similar regardless of source material (electronic supplementary material, tables S2 and S6). Also, as described in Mann *et al.* [71], we note that the average fragment length of endogenous human DNA was longer in dentine than in the calculus (70 versus 51 bp). In addition, we found that *M. leprae* DNA was slightly longer in the dentine sample compared to the calculus (59 in dentine versus 46 bp; electronic supplementary material, table S2). Notably, each substrate was sequenced on different platforms (BGIseq500 for calculus and Illumina for dentine); however, previous comparisons have found no significant differences between the two platforms both for ancient metagenomic and metatranscriptomic data [24,72] or for modern microbial data [73].

*Mycobacterium leprae* is well known for its thick cell wall [14,74–77] which may explain the observed similarity in deamination rates and fragment lengths for *M. leprae* DNA in calculus and dentine. The protective nature of calculus and the mycobacterial cell wall may also explain the low deamidation rates observed in the *Mycobacterium* peptides (electronic supplementary material, table S7). The variability in protein preservation with dental calculus has been previously reported [78] and the relatively young age of the sample may also explain the low deamidation rates.

## 4. Discussion

The presence of *M. leprae* DNA and peptides detected in the calculus suggests an oral manifestation in SK92, likely in the mucosa or soft palate, which led to *M. leprae* becoming entrapped during dental plaque formation. Overall, the reaction within the oral cavity in leprosy has been observed in 19–60% of clinical cases [79,80]*.* Irrespective of the presence of a leprous oral lesion, the oral cavity has been shown to harbour *M. leprae* bacteria even with a lack of specific visible lesions [81]; however, further studies are required to clarify whether the oral presence of the pathogen suggests greater severity or prolonged infection severity [82–84]. Poor dental and periodontal health have been associated with leprosy patients [85], and it was very likely that individual SK92 suffered from poor oral hygiene (electronic supplementary material, table S8 and figure S2). However, the possibility of any oral lesion (beyond the slight alveolar resorption noted above) is hard to discern from the osteological remains due to their preservation. Even in osteological remains with clear leprosy lesions, non-specific oral reactions are not always present [86]. The existence of *M. leprae* in the tooth-root dentin may be a result of the pathogen's presence in the blood or nerves inside the tooth root.

SK92 is the first ancient *M. leprae* genome recovered from Trondheim and, to our knowledge, from Norway overall. Leprosy was not uncommon during the sixteenth century in Trondheim, with evidence suggesting leprosy patients made up to 50% of applicants for admittance to hospitals during the fourteenth to seventeenth centuries [87,88]. Moreover, several other individuals with osteological evidence of leprosy have been recorded at the Library Site cemetery, as well as neighbouring contemporary cemeteries [88–90]. Notably, the oldest known hospital in Norway, founded in the twelfth century, was connected to Nidaros Cathedral, the largest religious institution in Trondheim. In the following centuries, the hospital was reorganized and relocated outside of the cathedral area [87] and was still active until the middle of the nineteenth century, with many of the patients recorded as leprosy patients throughout its use [87,88].

The presence of the SK92 *M. leprae* strain type in Norway during the sixteenth century (median probability is 1507 CE; see Results) extends the European distribution of branch 3I strains beyond Denmark and the UK during the Medieval period. SK92 is part of a multifurcation formed with the ancient SK2 (thirteenth century) genome and a branch leading to modern strains isolated from humans across the Americas, as well as one armadillo strain from the southwestern USA [49,68] (figure 2). The shared ancestry of these ancient European strains in relation to New World strains is not surprising, since leprosy is widely considered to have originated in the Old World, and no known evidence of pre-contact leprosy in the Americas is known so far [47,68,91]. The broad diversity of Brazilian strains in branch 3I is thought to originate from multiple European introductions of the 3I strain type to the Americas [49].

## 5. Conclusion

Our study is the first to demonstrate the recovery of ancient *M. leprae* biomolecules from archaeological dental calculus. We, therefore, highlight that it may represent an alternative sample source to bones and teeth, for detecting and molecularly characterizing *M. leprae* in individuals from the archaeological record, especially in the absence of definitive osteological markers. The robust nature of dental calculus increases its likelihood to be preserved over long periods of time. We hope it will prove to be a valuable substrate for future ancient pathogen studies to detect and retrieve biomolecular information for *M. leprae*, as well as other acquired infectious pathogens that are present in the oral cavity during disease progression. This is particularly relevant for cases where human remains are poorly preserved or too precious to warrant destructive bone sampling.

## Supplementary Material

Supplementary Figures 1-3

## Supplementary Material

Supplementary Tables 1-8

## Data accessibility

The mass spectrometry proteomics data were submitted to the ProteomeXchange Consortium via the PRIDE [92] partner repository with the dataset identifier PXD017836. Sequence data that support the findings of this study have been submitted to the European Nucleotide Archive under accession number PRJEB37995. All other data supporting the findings of this study are available upon request.
