# Supplementary material for: Multi-omic detection of Mycobacterium leprae in archaeological human dental calculus
Source: Philos Trans R Soc Lond B Biol Sci. 2020 Oct 5;375(1812):20190584. doi: 10.1098/rstb.2019.0584 (PMC7702802; doi:10.1098/rstb.2019.0584)
Supplement: Supplementary Figures 1-3 [file rstb20190584supp1.pdf]

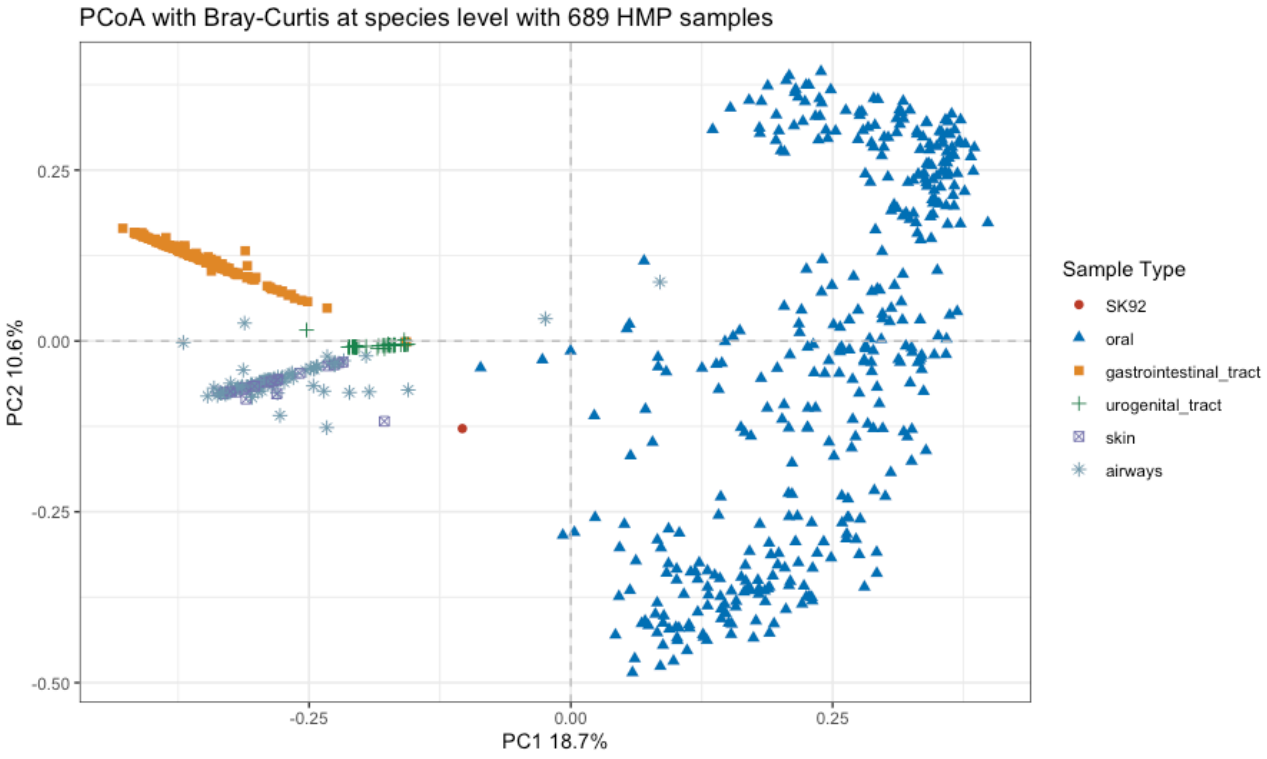

Supplementary Figure 1. PCoA comparison between species identification of the Medieval calculus sample (SK92 in red) and the HMP dataset.

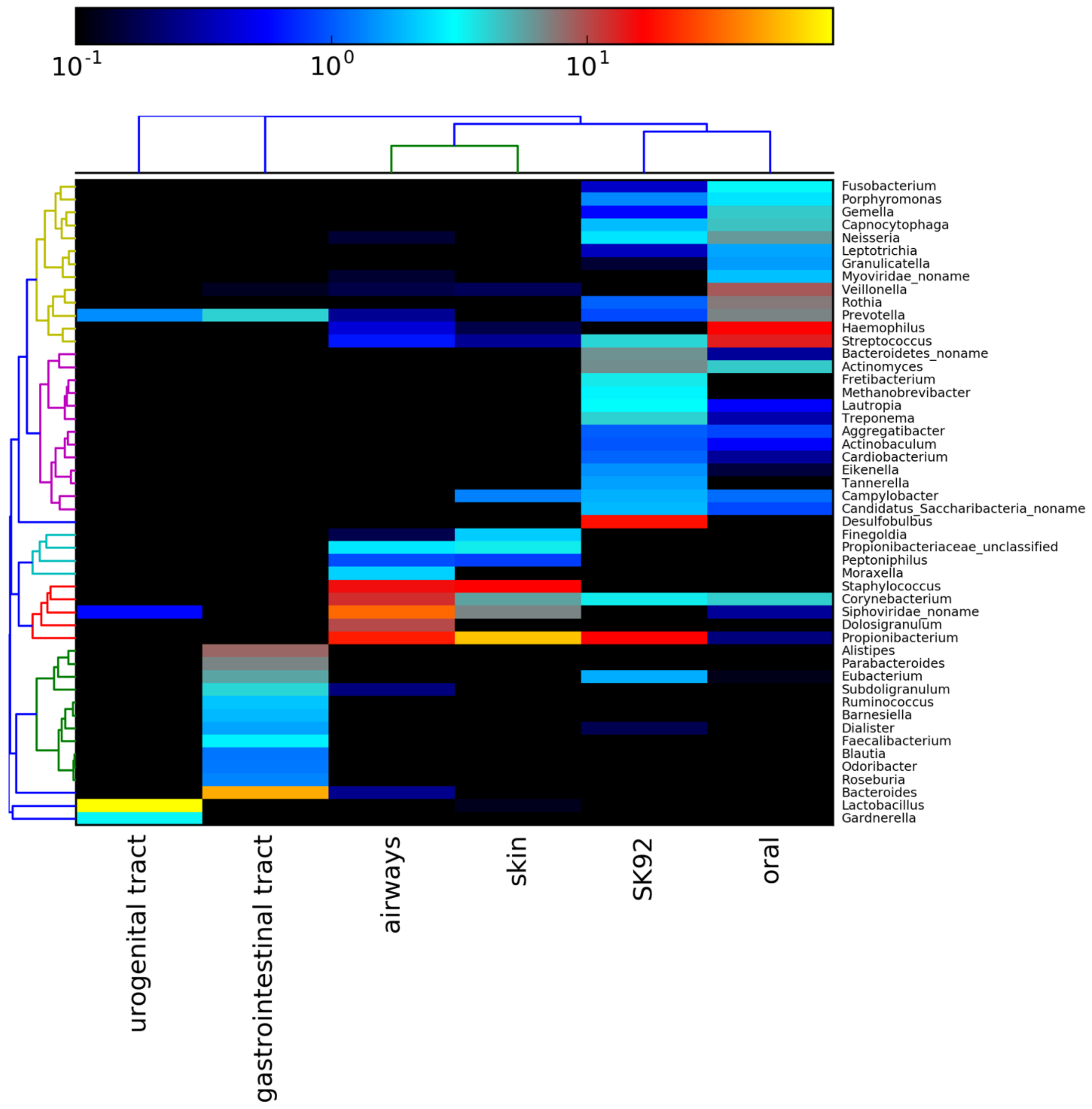

Supplementary Figure 2. Top 50 genera identified in the different human body sites from the Human Microbiome Project and the SK92 calculus sample. In red the most abundant genera.

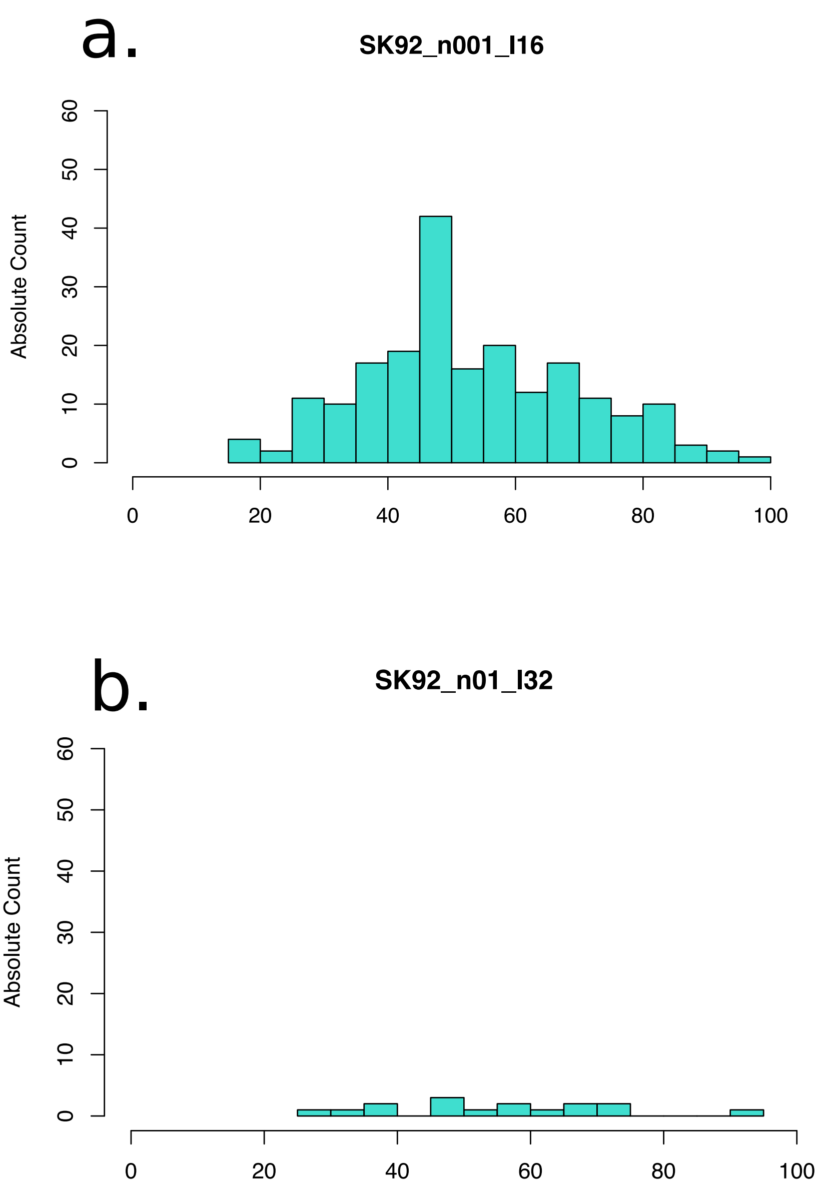

Supplementary Figure 3. Histograms of SNP allele frequency distributions for the a) sensitive (-n 0.01, -l 16) and b) stringent (-n 0.1, -l 32) mapped versions of SK92 calculus *M. leprae* genome. The x-axis shows the SNP allele frequencies as a percentage. All variants where the SNP allele frequency is higher than 10% and lower than 100% are shown.
